# Living condition, weight loss and cognitive decline among people with dementia

**DOI:** 10.1002/nop2.137

**Published:** 2018-03-23

**Authors:** Marcela Harsányiová, Pavol Prokop

**Affiliations:** ^1^ Faculty of Health Care and Social Work Trnava University Trnava Slovakia; ^2^ Faculty of Education Department of Biology Trnava University Trnava Slovakia; ^3^ Institute of Zoology Slovak Academy of Sciences Bratislava Slovakia

**Keywords:** Alzheimer's disease, BMI, cognitive performance, loneliness, vascular dementia

## Abstract

**Aims:**

The aim of this study was to investigate cognitive performance and BMI of patients with dementia living in their own homes with family members, nursing homes and alone.

**Design:**

A prospective observational cohort study with a quantitative design.

**Method:**

Mini–mental state examination (MMSE) scores and BMI were examined with a sample of Slovak patients (*N* = 428). Patients were followed up 12 months later after the first examination.

**Results:**

Cognitive decline was significantly faster for patients living in nursing homes and for solitary patients. BMI consistently decreased in the follow‐up examination and this drop was stronger in patients living alone and in nursing homes. Patients with VaD manifested a stronger BMI decline as compared with AD patients. This study suggests that impoverished conditions such as nursing homes or social isolation of solitary people contribute to stronger progress in dementia. Healthcare professionals need to implement meaningful activities for institutionalized people and for people who are living alone to eliminate the negative impact of an impowerished environment on patient's cognitive functioning.

## 1 INTRODUCTION

Dementia is a progressive, degenerative brain disease causing a loss of cognitive and emotional functioning (Cypriani, Lucetti, Carlesi, Danti, & Nuti, 2015). Estimates suggest that there are about 24 million people living with dementia worldwide and that this number will be three or four times higher by 2040 (Ferri et al., 2005). Epidemiological studies on dementia prevalence in Slovakia are missing, but it is estimated that at least 50–60,000 people live with Alzheimer's disease, the most common form of dementia (Slovak Alzheimer's Society 2010).

Nursing homes (NH) are very common settings for care for individuals with dementia; 90% of individuals with dementia are cared for in a nursing home and as many as 70% will die there (Givens, Lopez, Mazor, & Mitchell, 2012; Mitchell et al., 2005; Teno et al., 2004). The remaining individuals obviously die at home with or without additional nursing care (Kay, Forster, & Newens, 2000; Macdonald & Cooper, 2007).

### 1.1 Background

Families with NH residents often report inadequacies in patient care, physician communication, tube feeding, lack of privacy and emotional support (Biola et al., 2007; Engel, Kiely, & Mitchell, 2006; Givens et al., 2012; Teno et al., 2004; Wetle, Shield, Teno, Miller, & Welch, 2005). Carlson's (2007) and Wowchuk, McClemet, and Bond's (2007) reviews of published articles additionally revealed that health workers lack adequate knowledge and skills. There is a frequent absence of physicians in nursing homes, a lack of attention to symptom control and spiritual needs, poor hygiene and lack of care planning. These variables are in all probability responsible for lower family satisfaction with nursing homes compared with home or hospital settings (Teno et al., 2004). Furthermore, NH can be a source of psychological stress to their residents; more than 80% of NH staff in the USA observed at least one psychologically abusive incident (Pillemer & Moore, 1989) and 79% of NH staff surveyed in Germany confessed that they had abused or neglected a resident at least once over the previous 2 months (Goergen, 2001).

Psychological stress can negatively affect cognitive function both when short‐ and long‐term stressors are considered (Scott et al., 2015; Sliwinski, Smyth, Hofer, & Stawski, 2006; Stawski, Sliwinski, & Smyth, 2006). Long‐term, chronic stress has been associated with a poorer cognitive function (Andel, Crowe, Kareholt, Wastesson, & Parker, 2011; Korten, Sliwinski, Comijs, & Smyth, 2014), accelerated cognitive decline (Aggarwal et al., 2014; Wilson et al., 2005) and an increased incidence of dementia (Wilson, Arnold, Schneider, Li, & Bennett, 2007). Moving to a nursing home where the older people no longer permanently live with their relatives is stressful for them (An & Jo, 2009; Givens et al., 2012). Furthermore, institutionalization aggravates cognitive decline, probably due to the impoverished environment of nursing homes, defined by Volkers and Scherder (2011) as an environment with limited possibilities for physical and social activity (González‐Colaço Harmand et al., 2014; Scocco, Rapattoni, & Fantoni, 2006; Wilson, McCann, et al., 2007; Winocur & Moscovitch, 1990). Indeed, a sample of research revealed a negative association between social isolation and cognitive functioning (Conroy, Golden, Jeffares, O'Neill, & McGee, 2010; O'Luanaigh et al., 2012; Tilvis et al., 2004). A cognitive decline was also documented in non‐human animals, particularly in older individuals (for a review see Volkers & Scherder, 2011). Old rats, for example, living in an impoverished environment for 92 days showed a decline in learning and memory compared with rats in a standard environment (Winocur, 1998). The same can be applied to sedentary and lonely people who have, due to an impoverished environment, worse cognitive functions and faster cognitive decline than physically and socially active people (Ayalon, Shiovitz‐Ezra, & Roziner, 2016).

Living in nursing homes (Bauer et al., 2008; Donini et al., 2013; Stange, Poeschl, Stehle, Sieber, & Volkert, 2013; Suominen et al., 2005) and the presence of dementia (Barrett‐Connor, Edelstein, Corey‐Bloom, & Wiederholt, 1996; Gillette‐Guyonnet et al., 2007) are major factors in weight loss. Malnutrition is linked to considerable harmful consequences such as increased mortality (Morley, 2003), impairment in immune responses (Lesourd, 1997), increased risk of hip fracture (Ensrud, 2003), incontinence (Rose, Thimme, Halfar, Nehen, & Rübben, 2013), sleep disturbances (Yildiz, Pekel, Kilic, Tolgay, & Tufan, 2015) and rapid cognitive decline in persons with Alzheimer's disease (Soto et al., 2012, Yildiz et al., 2015). Koyama et al. (2016) recently demonstrated that malnutrition in patients with dementia and AD is associated with biochemical blood markers such as low levels of haemoglobin and serum albumin. Analyses of data from 12 countries revealed that the prevalence of malnutrition in NH was 13.8% (Kaiser et al., 2010). Several authors have demonstrated that loneliness is also related to dietary inadequacies (Walker & Beauchene, 1991) and undernutrition (Pilrich & Lochs, 2001; Nogay & Akıncı, 2012; Eskelinen, Hartikainen, & Nykänen,^2016^). We suggest that body mass index (BMI) will drop amongst solitary people and those living in NH more rapidly as compared with people living with families.

### 1.2 Aim of the study

The aim of this study is twofold. First, we examined the cognitive performance of patients with vascular dementia (VaD) and Alzheimer's disease (AD) with respect to three different living conditions (homes with family members, nursing homes and alone). We hypothesize that residents living in nursing homes and alone have a worse cognitive performance compared with residents living in their homes. Secondly, we examined possible differences in BMI in these patients with respect to the same conditions to discover whether weight loss is faster in NH residents or in solitary patients compared with those living in homes with their families.

## 2 THE STUDY

### 2.1 Design

This prospective observational cohort study adopted a quantitative research design.

### 2.2 Sample

The findings were derived from data collected through two waves of in‐depth, open‐ended examinations conducted over the course of the years 2011–2014. A total of 681 patients from predominantly the Bratislava region, Slovakia, visiting the private surgery of the first author of this study comprised the sample. A significant part of them (*N = *253) were removed from analyses because they died before the second examination, or because they suffered from various additional health problems (e.g. hypertension, cachexia) to make the sample as homogeneous as possible. The mean age of the patients (*N = *269 [63%] were women) was 77.4 years (*SD* 4.47) with a range of 66–91 years (*N = *428). All the patients were diagnosed with VaD (*N = *217, 51%) or AD (*N = *211, 49%).

### 2.3 Data collection

We examined patients two times each at approximately 12‐month intervals. All the examinations were performed by the same person (MH).

#### 2.3.1 Weighing

Each patient first undressed down to their underwear and was weighed on a digital certificated scale (OMRON HN 288) to the nearest ±0.1 kg during each examination. All patients were weighed on the same scale. The height of the patients was measured to the nearest 1 cm during the first examination.

#### 2.3.2 Cognitive performance

All the patients were examined with a mini–mental state examination (MMSE) 30‐point questionnaire which is used extensively in clinical and research to screen for dementia (e.g., Meaney, Croke, & Kirby, 2005; Mortimer, Ebbitt, Jun, & Finch, 1992; Pangman, Sloan, & Guse, 2000). Repeated use of the MMSE in patients with diagnosed dementia was also reported by other authors (e.g., González‐Colaço Harmand et al., 2014; Samuel et al., 2000; Scocco et al., 2006). The test administration obviously did not exceed 10–30 min. Higher MMSE scores indicate a better cognitive performance. The mean MMSE scores from the first examination were almost always higher than the scores from the second examination. This suggests that patient's cognitive performance declined. A high difference meant that the progress in dementia was rapid while a low difference suggested that the progress in dementia was slower.

### 2.4 Data analysis

Differences in the MMSE scores between the patients (dependent variable) living in NH, with families and solitary (categorical predictor), were compared with the general linear model (GLM) with patients’ age defined as covariate. Mean difference scores from the MMSE questionnaire (first minus second examination) were not normally distributed (Kolmogorov–Smirnov test, d = 0.15, *p *<* *.001) and advanced examination of the data revealed that they corresponded to the Gamma distribution. This is a two‐parameter family of continuous probability distributions. More details about parameters can be found in Boland (2007, p. 43). In this case of distribution, however, all data should have higher values than zero. In our case, two patients (0.5%) showed no differences in the first and second examination scores (i.e., their final score was 0); thus, we used X + 0.1 transformation of the data to obtain positive values. The data were finally analysed with a generalized linear model with gamma distribution. The categorical predictors in the model were gender, type of care and type of dementia with age defined as covariate. Differences between the means were subsequently carried out with analysis of contrasts. Non‐significant interaction terms were removed, and the model was run again (according to Zuur et al., 2009). Regarding potentially confusing variables (relationship status, education, having children or not), their inclusion into the statistical models did not change the results presented below.

Differences between the first and second examination of BMI always resulted in negative values; thus, we could not employ the same approach as with the data from the questionnaire. Data on BMI were Box–Cox transformed and normality was achieved. The general linear model where BMI was measured during the first and second examinations was consequently defined as the within‐subject variable. Categorical predictors in the model were gender, type of care and type of dementia while age was defined as covariate. Differences between means were examined with the Tukey post hoc test. Statistical tests were performed with the software Statistica (Version 8, StatSoft 2007, Tulsa, Oklahoma, USA, http://www.statsoft.com).

### 2.5 Ethical considerations

Ethics approval was obtained from the Faculty of Health Care and Social Work, Trnava University.

## 3 RESULTS

### 3.1 Descriptive statistics of the sample

36 (8%) of the patients were single, 308 (72%) were widowed, 26 (6%) were divorced and 58 (14%) were married. 367 (86%) of the patients had at least one child, the remaining were childless. Most of the patients had completed primary school (*N = *218, 51%), less secondary school (*N = *108, 25%) while the remaining 102 patients (24%) had completed university. The majority of the patients (*N = *195, 45%) lived for <30 days in nursing homes before the first examination, less in their own homes with family members (*N = *153, 36%) while the remaining 80 patients (19%) were alone. Further details regarding differences between groups can be found in Table 1.

**Table 1:** Baseline characteristics of patients living in three different conditions

	Family	Nursing home	Alone	Test value^a^
Age	79.6 (*SD* 5.22)	76.1 (*SD* 2.93)	76.5 (*SD* 4.43)	32.9***
Sex (women): *N*(%)	84 (55)	138 (71)	47 (59)	9.9**
Relationship status: *N*(%)				32.95***
Single	15 (42)	15 (42)	6 (16)	
Widowed	87 (28)	159 (52)	62 (20)	
Divorced	14 (54)	9 (35)	3 (11)	
Married	37 (64)	12 (21)	9 (15)	
With children: *N* (%)	134 (37)	159 (43)	74 (20)	6.23*
Education: *N* (%)				17.5**
Primary	90 (41)	90 (41)	38 (18)	
Secondary	26 (15)	51 (47)	31 (38)	
University	37 (36)	54 (53)	11 (11)	

a Probability values are determined using the χ2 test or by ANOVA as appropriate.

*p < .05, **p <* *.01, ***p <* *.001.

### 3.2 Basic differences in MMSE test scores

Table 2 indicates mean MMSE scores for three groups of patients. GLM with the MMSE score from the first examination as the dependent variable revealed that the scores significantly differed between the three groups of patients (*F*(2,42) = 5.94, *p *=* *.003). Solitary patients had significantly lower mean scores than patients living in NH (Tukey post hoc test, *p *=* *.02), but other differences were not significant (all *p *>* *.24). Differences in the follow‐up examination were also statistically significant (GLM, *F*(2,42) = 30.4, *p *<* *.001). Patients living with their families scored significantly better than patients from NH and patients living alone (Tukey post hoc tests, all *p *<* *.01). The latter group of patients scored lower than patients from NH (Tukey post hoc test, *p *<* *.001).

**Table 2:** Descriptive statistics for the MMSE scores (means ± SD) in three groups of patients obtained in the first and second examinations

	Family	Nursing homes	Alone
First examination	25 (*SD* 2.43)	25.4 (*SD* 3.26)	24.4 (*SD *3.34)
Second examination	21.1 (*SD* 4.11)	19.2 (*SD* 6.67)	14.2 (*SD* 6.91)

### 3.3 Differences in MMSE test scores after 12 months

**Figure 1:**
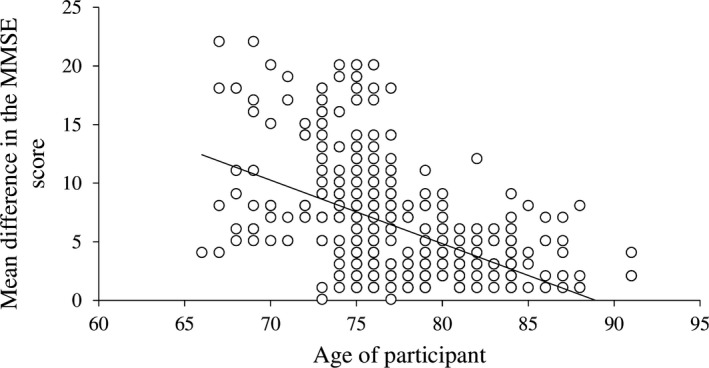
Association between the patient's age and differences in the MMSE score. Younger patients received higher first–second examination score differences suggesting that dementia had faster progress

The Cronbach's alpha of the MMSE questionnaire for the first‐ and second‐examination phase was high (0.71 and 0.90 respectively). The test–retest reliability was also acceptable (Guttman split‐half reliability = 0.67, Spearman–Brown coefficient = 0.79), indicating that measurements during both the first and second examinations were reliable. The patient's age was significantly associated with the final MMSE score meaning that progress in dementia was more rapid amongst younger patients compared with older patients (Table 3, Figure 1). Gender did not influence the final MMSE score.

**Table 3:** Results on a generalized linear model with gamma distribution on the MMSE score. All independent variables except for gender significantly influenced the MMSE score

	Level of effect	Column	Estimate	Wald	−95% CL	+95% CL	*p*
Intercept		1	7.8	186.8	6.7	8.9	<.0001
Gender	Female^a^	2	−0.04	1.2	−0.1	0.03	.27
Type of care	Family^b^	3	−0.3	32.3	−0.4	−0.2	<.0001
Type of care	NH^c^	4	−0.1	6.2	−0.2	−0.02	<.05
Type of dementia	AD^d^	5	−0.2	23.5	−0.2	−0.1	<.0001
Type of care × Type of dementia	1	6	−0.2	12.4	−0.2	−0.07	<.001
Type of care × Type of dementia	2	7	0.1	6.04	0.02	0.2	<.05
Age		8	−0.08	111.6	−0.09	−0.06	<.0001
Scale			2.5		2.2	2.9	

a Females are compared with males.

b Patients living in families are compared with patients living in nursing homes.

c Patients living in nursing homes are compared with solitary patients.

d Patients with Alzheimer's diagnoses are compared with patients with vascular diagnoses.

**Figure 2:**
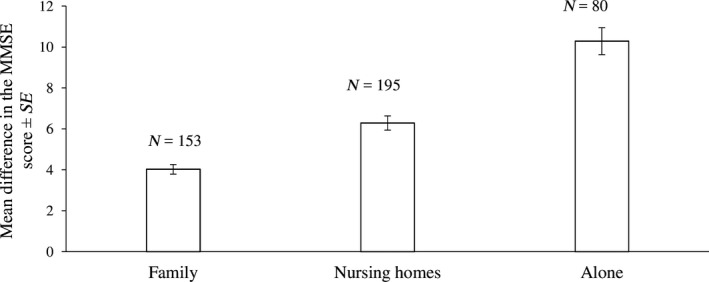
Differences in the MMSE score (weighed marginal means) with respect to the type of care

Patients who were in family care showed significantly slower progress in dementia compared with patients in dementia centres and solitary patients (analysis of contrasts, all *p *<* *.001, Table 1, Figure 2). Solitary patients manifested the fastest progress in dementia scores and patients in dementia centres received intermediate scores.

The type of dementia was also associated with the final MMSE dementia scores (Table 3). Patients with Alzheimer's diagnoses in particular (weighed mean = 5.55 *SD *4.76, *N = *211) showed slower progress in dementia MMSE final scores compared with patients with vascular diagnoses (weighed mean = 6.87 *SD *5.11, *N = *217).

**Figure 3:**
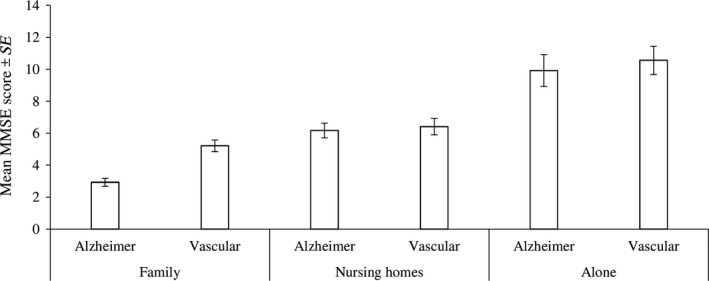
Descriptive statistics for distribution of the MMSE score among patients with respect to the type of care and type of dementia

When considering the interaction terms between the variables (Table 3), the type of care × type of dementia interaction suggests that under family care, patients with Alzheimer's diagnosis scored lower (i.e., the progress in dementia was slower) than patients with vascular diagnoses (Figure 3).

### 3.4 Differences in BMI

**Figure 4:**
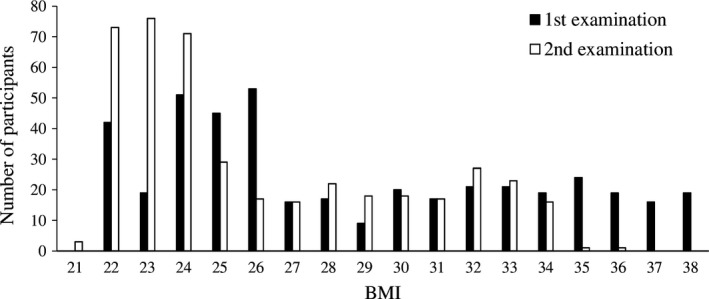
Descriptive statistics for distribution of BMI among patients across two examinations

BMI ranged between 21 and 38 (Figure 4), suggesting that none of the patients were underweight. With regard to between‐subject variables, gender differences (BMI of males and females during first examination, mean *=* 28.63, *SE *0.22, *N = *159 and mean* = *28.64, *SE *0.18, *N = *269 respectively) were not statistically significant (*F*(1,415) = 0.6, *p *=* *.46). Family care (BMI during first examination, mean = 28.25, *SE* 0.21, *N = *153) and care in nursing homes (BMI during first examination, mean* =* 28.6, *SE* 0.21, *N = *195) were associated with lower and non‐significantly different BMIs (Tukey post hoc test, *p *=* *.9), while patients who lived alone showed higher BMI (BMI during first examination, mean = 29.1, *SE *0.3, *N = *80) (*F*(2,415) = 4.3, *p *=* *.01) than patients from the former two groups (Tukey post hoc test, both *p *<* *.001). Patients with Alzheimer's diagnoses manifested lower BMI (BMI during first examination, mean = 24.33, *SE* 0.2, *N = *211) compared with patients with vascular diagnoses (BMI during first examination, mean = 32.95, *SE* 0.2, *N = *217) (*F*(1,415) = 1146.3, *p *<* *.0001). Older patients tended to have slightly higher BMI than younger patients (*F*(1,415) = 5.9, β = 0.06, *p *=* *.02). The interaction terms were not statistically significant (all *p* ˃ .07).

With regard to within‐subject variables, BMI was significantly lower in the second (mean = 26.08, *SE *0.24) compared with the first examination (mean = 28.58, *SE *0.19) (*F*(1,415) = 56380.0, *p *<* *.0001). Patients with vascular diagnoses showed a stronger drop in BMI between the first and second examination (difference in BMI, mean = −3.5, *SE* 0.25, *N = *217) compared with patients with Alzheimer's diagnoses (difference in BMI, mean = −1.45, *SE* 0.26, *N = *211) (*F*(1,415) = 1140.6, *p *<* *.0001). Family care resulted in the lowest change in BMI (difference in BMI, mean = −2.19, *SE* 0.28, *N = *153), while patients in nursing homes (difference in BMI, mean = −2.69, *SE* 0.25, *N = *195) and those who lived alone (difference in BMI, mean = −2.53, *SE* 0.25, *N = *195) showed a stronger drop in their BMI (*F*(2,415) = 4.3, *p *=* *.01). BMI drop was stronger in older patients compared with younger patients (*F*(1,415) = 5.9, *p *=* *.02). Additional interaction terms were not statistically significant (all *p *>* *.07).

## 4 DISCUSSION

Using standardized measures, we examined cognitive performance and changes in BMI in patients with VaD and AD. We found a significant drop in the cognitive performance measured by the MMSE with respect to environmental conditions, but no evidence regarding malnutrition was observed.

Our first hypothesis dealt with possible differences in the MMSE based on the three conditions where the patients live: homes with family members, nursing homes and alone. The cognitive performance of all but two patients decreased in all probability as a function of dementia progression (Cypriani et al., 2015). In line with our hypothesis, patient's cognitive performance decreased significantly more when they lived alone or in nursing homes compared with patients living in homes with their families. This finding is particularly important because a majority of dementia patients in western countries live in nursing homes (Givens et al., 2012; Mitchell et al., 2005; Teno et al., 2004). Our sample revealed a similar trend since, compared with solitary patients and those living at home, a majority of patients (45%) lived in nursing homes.

To date, few studies have shown that living in nursing homes is associated with cognitive decline (González‐Colaço Harmand et al., 2014; Scocco et al., 2006; Wilson, McCann, et al., 2007; Winocur & Moscovitch, 1990). The results of these studies suggest that the transition from the community to a nursing home is cognitively deleterious. However, these findings can potentially be explained by pre‐existing differences between patients, since dementia is one of the main risk factors associated with institutionalization (Gnjidic et al., 2012), González‐Colaço Harmand et al. (2014) showed that even after exclusion of the patients who developed dementia during the follow‐up examination, their results indicating a cognitive decline as a result of institutionalization remained unchanged. Furthermore, our sample only consists of dementia patients suggesting that the risk of being institutionalized was similar for all the patients. Negative emotional states caused by social isolation and insufficient physical activity are associated with stress and higher concentrations of cortisol, a stress hormone, which is related to the impaired cognitive function (Fratiglioni, Paillard‐Borg, & Winblad, 2004). Alternatively, the rapid decline of cognitive functions can later cause social isolation, as it was recently suggested by Ayalon et al. (2016). Long‐term examination data are required to test these two possibilities.

Loneliness is associated with limited social networks and social engagements and is consequently associated with a faster cognitive decline in older people with or without cognitive impairment (Andrew & Rockwood, 2010; Bennett, Schneider, Tang, Arnold, & Wilson, 2006; Cacioppo & Hawkley, 2009; Fratiglioni et al., 2004; Karp et al., 2006; Krueger et al. 2009). In line with our findings, Meaney et al. (2005) found that lower MMSE scores were associated with loneliness. Furthermore, Newall, Chipperfield, Bailis, and Stewart (2013) revealed that loneliness is an independent risk factor for mortality and reduced physical activity among older adults. Low physical activity (Hawkley, Thisted, & Cacioppo, 2009), unmet social and emotional demands (Meaney et al., 2005), an impoverished environment (Volkers & Scherder, 2011) and probably an absence of care from family members associated with lower emotional support (Ellwardt, Aartsen, Deeg, & Steverink, 2013) may negatively influence both the emotional and cognitive performance of solitary people.

The second aim of this research was to compare BMI between patients living in NH, alone and in their own homes with family members. Patients with VaD showed a stronger decline in BMI over 12 months compared with patients with AD. This result can be explained by more frequent dietary restrictions recommended by physicians to reduce the incidence of ischaemic heart disease and stroke in patients with VaD (Zimetbaum, Frishman, & Aronson, 1991). AD patients frequently suffer from appetite loss (Finkel, 2000; Finkel, Costa E Silva, Cohen, Miller, & Sartorius, 1996) which can explain the BMI drop in this study.

Both weight loss and cognitive decline can be in mutual association; weight loss is one of factors included in the operational definitions of frailty in older age, a syndrome particularly common in nursing homes (Kaiser et al., 2010). Given that cognitive impairment and dementia are causally linked with frailty (Panza et al., 2015); it seems that weight loss and/or other forms of physical frailty are associated with the development of dementia and AD (Panza et al., 2015).

Patients who lived alone showed the highest BMI compared with patients from the remaining two groups. These results are surprising at first glance because loneliness is obviously associated with malnutrition (Donini et al., 2013; Ramic et al., 2011; Shahar, Shai, Vardi, & Fraser, 2003). We suggest that their diet is not under a similar control as with patients in nursing homes or with families. It can therefore consist of high calories although unhealthy foods may promote high BMI (Neumark‐Sztainer, Wall, Story, & Standish, 2012). Perceived social isolation and a negative affect are associated with binge eating (Mason, Heron, Braitman, & Lewis, 2016) which can also contribute to higher BMI in solitary patients. It should be noted that food intake amongst solitary people depends on a great extent on their attitudes towards cooking (Hughes, Bennett, & Hetherington, 2004), distance from food markets and the patient's socio‐economic status (Donini et al., 2013). These variables were not examined in this study but could at least partly influence the presented results.

Family care was associated with the smallest BMI decline compared with patients living in nursing homes or alone. The diet preservation recommended by physicians may be significantly lower amongst these patients compared with the strict conditions in nursing homes. There is no data, however, to support this claim although our personal experiences strongly support this possibility (M. Harsányiová, personal experiences). In this case, the lower drop in BMI needs not have positive outcomes on the health of patients. An increased decline in BMI in patients from nursing homes could be, conversely, influenced by inadequate care in nursing homes (Donini et al., 2013; Stange et al., 2013). This latter claim can be supported by a comparable major BMI drop amongst solitary people, who are unable to obtain and cook foods of the required amount and quality (Donini et al., 2013; Hughes et al., 2004; Ramic et al., 2011). Further in‐depth research involving family members is clearly required to support our hypothesis.

### 4.1 Limitations

The main limitation of this study is lack of patients living alone. Unfortunately, most of family members or neighbours are not interested in this cohort of people, which finally resulted in smaller sample size. Secondly, we have not enough childless patients who deserve special attention. According to our own experiences, childless patients who have lived for a long time alone enjoy NH more than those who have children. Childless patients obviously enjoy relatively rich social ties, thanks to the interest of other patients or staff in NH. We suggest that the investigation of NH from the perspective of psychosocial and health benefits for childless patients would be an avenue for further research.

## 5 CONCLUSION

Nursing homes and loneliness would seem to be statistically significantly associated with stronger progress in cognitive performance in patients with dementia. Correspondingly, BMI shows more rapid drop, particularly in patients living in nursing homes and in solitary patients. An increased emphasis should be placed on activities and social interactions in impoverished conditions represented by nursing homes or by solitary people living in social isolation. Further research should be directed at the food quality of dementia patients living under various conditions.

### 5.1 Relevance to clinical practice

Patients in NH should be provided with a daily programme with diverse activities to stimulate cognitive functions. Physical activities (e.g., rehabilitation, working therapy and physical training) should be planned for various parts of the day, to prevent physical decline. Patients should live with other patients who are at a similar level (not more progressive) of dementia and, if possible, rooms should be equipped with furniture from their homes.

In case of patients living alone, family members and/or social authorities should be contacted and trained to provide adequate home care for them. Social authorities should regularly monitor patients living alone and secure adequate health care and/or recommend any adequate NH.

If the patient is living with family members, they should also be educated to provide adequate health care and rehabilitation. Any personal changes in the care for dementia patients should only be done in terminal phases of dementia or if possible, not at all. Patients should not live in isolation but should be involved in common daily activities to stimulate their cognitive functions.

## CONFLICT OF INTEREST

Authors declare no conflict of interest.

## AUTHOR CONTRIBUTIONS

MH and PP were involved in the design of the study; MH and PP were collected and analysed the data; and MH and PP were prepared the manuscript.
